# A Panel of *Trypanosoma brucei* Strains Tagged with Blue and Red-Shifted Luciferases for Bioluminescent Imaging in Murine Infection Models

**DOI:** 10.1371/journal.pntd.0003054

**Published:** 2014-08-21

**Authors:** Nick Van Reet, Hélène Van de Vyver, Patient Pati Pyana, Anne Marie Van der Linden, Philippe Büscher

**Affiliations:** 1 Department of Biomedical Sciences, Institute of Tropical Medicine, Antwerp, Belgium; 2 Institute of Medical Microbiology, University Hospital of Münster, Münster, Germany; 3 Département de Parasitologie, Institut National de Recherche Biomédicale, Kinshasa Gombe, Democratic Republic of the Congo; 4 Faculty of Pharmaceutical, Biomedical and Veterinary Sciences, Bio-Imaging Lab, Department of Biomedical Sciences, University of Antwerp, Wilrijk, Belgium; New York University School of Medicine, United States of America

## Abstract

**Background:**

Genetic engineering with luciferase reporter genes allows monitoring *Trypanosoma brucei* (*T.b.*) infections in mice by *in vivo* bioluminescence imaging (BLI). Until recently, luminescent *T.b.* models were based on *Renilla* luciferase (RLuc) activity. Our study aimed at evaluating red-shifted luciferases for *in vivo* BLI in a set of diverse *T.b.* strains of all three subspecies, including some recently isolated from human patients.

**Methodology/Principal findings:**

We transfected *T.b. brucei*, *T.b. rhodesiense* and *T.b. gambiense* strains with either RLuc, click beetle red (CBR) or *Photinus pyralis* RE9 (PpyRE9) luciferase and characterised their *in vitro* luciferase activity, growth profile and drug sensitivity, and their potential for *in vivo* BLI. Compared to RLuc, the red-shifted luciferases, CBR and PpyRE9, allow tracking of *T.b. brucei* AnTaR 1 trypanosomes with higher details on tissue distribution, and PpyRE9 allows detection of the parasites with a sensitivity of at least one order of magnitude higher than CBR luciferase. With CBR-tagged *T.b. gambiense* LiTaR1, *T.b. rhodesiense* RUMPHI and *T.b. gambiense* 348 BT in an acute, subacute and chronic infection model respectively, we observed differences in parasite tropism for murine tissues during *in vivo* BLI. *Ex vivo* BLI on the brain confirmed central nervous system infection by all luminescent strains of *T.b. brucei* AnTaR 1, *T.b. rhodesiense* RUMPHI and *T.b. gambiense* 348 BT.

**Conclusions/Significance:**

We established a genetically and phenotypically diverse collection of bioluminescent *T.b. brucei*, *T.b. gambiense* and *T.b. rhodesiense* strains, including drug resistant strains. For *in vivo* BLI monitoring of murine infections, we recommend trypanosome strains transfected with red-shifted luciferase reporter genes, such as CBR and PpyRE9. Red-shifted luciferases can be detected with a higher sensitivity *in vivo* and at the same time they improve the spatial resolution of the parasites in the entire body due to the better kinetics of their substrate D-luciferin.

## Author Summary

Research on African trypanosomes heavily relies on rodent infection models. One way to reduce the number of laboratory rodents used in each experiment and effectively follow the progression of the infection in the same animals is to use genetically modified trypanosomes that allow monitoring of the infection over time with bioluminescence technology, without having to sacrifice the animals at multiple time points. In this study, we were able to establish a collection of bioluminescent strains of all three subspecies of *Trypanosoma brucei* (*T.b.*), including *T.b. gambiense* and *T.b. rhodesiense* that cause human African trypanosomiasis (HAT) or sleeping sickness. Making use of bioluminescence assays, we demonstrate the diversity of our collection in terms of *in vitro* and *in vivo* growth, drug sensitivity and *in vivo* parasite distribution, including central nervous system tropism. Growth characteristics and drug sensitivity are not affected by the genetic modification with luciferase reporter genes. Trypanosome strains transfected with red-shifted luciferase reporter genes have several advantages compared to the corresponding blue luciferase modified strains. Red light is less absorbed in the blood than blue light, which should lead to higher sensitivity of detection. Furthermore, the substrates that drive the light reaction are better distributed through the body for the red luciferase than for the blue luciferase, which greatly improves spatial resolution of the infection.

## Introduction

African trypanosomes pose a threat to millions of humans and animals in sub-Saharan Africa. Only two species readily infect humans and both are subspecies of *Trypanosoma brucei* (*T.b.*). *T.b. gambiense* is responsible for the chronic form of human African trypanosomiasis (HAT) in West and Central Africa and accounts for more than 97% of the near 10,000 sleeping sickness patients who are diagnosed and treated annually [1]. *T.b. rhodesiense* causes a more acute form of HAT in South and East Africa, only differing from a third non human-infective subspecies, *T.b. brucei*, by a single gene, *SRA*, that confers resistance against human serum [2]. All *T.b.* subspecies are transmitted by the bite of tsetse flies (*Glossina spss*). Upon injection into a vertebrate host, parasites multiply locally in the lesion and cause a blood, lymph and tissue infection, also called first stage disease. Later, the parasites invade the central nervous system (CNS) initiating the second stage of the disease. Untreated infections almost invariably have a fatal outcome that occurs after weeks to months in *rhodesiense* HAT and months to years in *gambiense* HAT [3]. Treatment is subspecies- and stage-specific [1]. First stage *gambiense* and *rhodesiense* HAT are treated with pentamidine and suramin respectively. The first line treatment for second stage *gambiense* HAT consists of nifurtimox-eflornithine combination therapy (NECT) while second stage *rhodesiense* HAT is still treated with melarsoprol. All current drugs used to treat HAT are toxic [4].

For obvious reasons, basic and applied research on trypanosomes and HAT, including drug resistance studies, highly benefit from the availability of *T.b. rhodesiense* and *T.b. gambiense* strains that have been recently isolated from patients with known treatment outcome and that along their isolation underwent only few *in vivo* and/or *in vitro* passages [5]–[8]. To this end, bloodstream form trypanosomes can be isolated from diverse patient specimens such as blood, lymph or cerebrospinal fluid (CSF). Generally, *T.b. brucei* and *T.b. rhodesiense* can be easily isolated in classical laboratory rodents such as mice and rats [9]. *T.b. gambiense*, on the other hand, is very difficult to isolate and often requires either susceptible rodent species [9]–[12] or severely immune-suppressed or –deprived hosts [13], [14]. Seldomly, isolation of bloodstream form *T.b. gambiense* parasites has been achieved by direct inoculation of *in vitro* medium containing feeder layer cells [5], [15].

Apart from isolation of trypanosome strains, rodent models for HAT are considered to be informative because they can reproduce the invasion of the central nervous system (CNS) [16], [17]. Thus, these rodent models are highly relevant for investigating drug discovery, drug resistance and treatment failure [18], [19]. Much like the clinical diversity seen in HAT [1], rodent models also reveal a very broad spectrum of pathology resulting from infection with diverse strains of the human pathogenic subspecies of *T.b.* [5], [20]–[27]. Genetic engineering of parasites has made it possible to monitor infections in living animals using biophotonic techniques, such as *in vivo* fluorescence and bioluminescence imaging [28], [29]. *In vivo* bioluminescent imaging (BLI) allows the tracking of luciferase-modified cells in living animals over time without the need to sacrifice them. This technique has been applied to study the spatio-temporal distribution of *T.b. brucei* and *T.b. gambiense* parasites in murine models and lead to the discovery of a testis tropism of *T.b. brucei* AnTaR 1, where the parasites are less accessible for trypanocides [30]. BLI also revealed that different *T.b. gambiense* strains can induce a variety of infections in mice, ranging from chronic to silent, thus mimicking the clinical diversity that is observed in HAT [5]. The former models made use of RLuc as the bioluminescent reporter [31], which, upon oxidation of its substrate coelenterazine, emits blue light (peak luminescence at 480 nm) that is readily absorbed by blood and other tissues. Alternative luciferases emitting light with longer wavelengths exist, such as luciferase enzymes from fireflies, click beetles and railroad worms that all use D-luciferin as substrate [32], [33]. Firefly luciferase (FLuc) appeared to sort into the glycosome of *T.b.* possibly disturbing ATP/ADP equilibrium upon activation [34]. Recently the well characterised *T.b. brucei* GVR 35 strain was modified with a firefly luciferase variant (LUC2), that emits yellow light (560 nm), and proved to be useful to shorten the follow-up period in studies on drugs that reach the CNS during the infection [29]. Also, studies with LUC2 modified *T. vivax* have shown that this trypanosome species, like *T.b.*, invades tissues including the CNS [35]. However, luciferases that emit light beyond 600 nm are potentially even more useful for *in vivo* imaging due to the fact that transmission of light through animal tissue increases greatly above this wavelength [36]. The synthetic click beetle red luciferase (CBR) from *Pyrophorus plagiophtalamus* and the thermostabilised PpyRE9 luciferase from *Photinus pyralis* both emit light around 617 nm [37]. These red-shifted luciferases have shown a potential to better resolve signals from deeper tissues than the original FLuc or to emit more stable luminescence than the LUC2 variant [38], [39]. Very recently it was shown that PpyRE9-tagged *T.b. brucei* GVR 35 parasites allow improved detection in BLI over LUC2-tagged *T.b. brucei* GVR 35 models [40].

In the present study we explored the use of red-shifted firefly luciferases CBR and PpyRE9 for bioluminescent imaging of all three subspecies of *T.b.*, including the human pathogens, in comparison with the existing RLuc strains in our collection. This collection consists of a set of four genetically and phenotypically very different trypanosome strains (Table 1). *T.b. brucei* AnTaR 1 is a pleomorphic strain from Uganda that causes a sub-acute infection in various mouse and rat strains [41], [42]. *T.b. rhodesiense* RUMPHI is a recently isolated strain from Malawi, which underwent only a few *in vivo* and *in vitro* passages and originates from an area with less virulent *T.b. rhodesiense* than those circulating in Uganda [43], [44]. *T.b. gambiense* LiTaR 1 is a well characterised virulent strain that is used for the production of diagnostic antigens in the card agglutination test for trypanosomiasis (CATT) and in the recently developed HAT-Sero-*K*-SeT [45], [46]. This strain is extensively passaged *in vivo* and produces an acute monomorphic infection in rodents. In contrast, *T.b. gambiense* 348 BT was isolated in the HAT focus of Mbuji-Mayi in the Democratic Republic of the Congo (DRC) [7], where a very high relapse rate after melarsoprol treatment has been observed that may be related to the presence of a chimeric aquaglyceroporin 2/3 gene [47], [48].

**Table 1:** List of *T.b.* strains and their history.

Taxon	Strain	Original host	Country and HAT focus	Year	Passages in rodent	*In vitro* medium	Reference
*T.b. brucei*	AnTaR 1	bushbuck	Uganda, Busoga	1966	10	HMI	[77]
*T.b. rhodesiense*	RUMPHI	human	Malawi, Rumphi	2007	3	HMI	[51]
*T.b. gambiense*	LiTaR 1	human	Côte d'Ivoire	1967	14	HMI+ human serum	[78]
*T.b. gambiense*	348 BT	human	DRC, Mbuji-Mayi	2007	3	HMI+ human serum	[51]

We focus this report on the *in vitro* drug sensitivity, the *in vitro* growth characteristics and the *in vivo* virulence in mice, assessed through bioluminescence imaging of either RLuc, CBR and PpyRE9 luciferase activity, of the four bioluminescent *T.b.* strains.

## Materials and Methods

### Ethics statement

This study was approved by the Veterinary Ethics Committee of the Institute of Tropical Medicine, Antwerp, Belgium (protocol BM2012-1 and BM2013-5) and the Veterinary Ethics Committee of the University of Antwerp, Belgium (protocol BPI-EAT). It adheres to the European Commission Recommendation on guidelines for the accommodation and care of animals used for experimental and other scientific purposes (18 June 2007, 2007/526/EG) and the Belgian National law on the protection of animals under experiment. The parasite strains included in this study belong to the cryobank of the World Health Collaboration Center for Research and Training on Human African Trypanosomiasis Diagnostics at the Institute of Tropical Medicine in Antwerp, Belgium.

### *Trypanozoon* strains and *in vitro* culture

The axenic *in vitro* culture of monomorphic and pleomorphic bloodstream form trypanosome populations in HMI-9 has been described elsewhere [49], [50]. The original host, the year and country of isolation, the number of *in vivo* passages and the medium for *in vitro* culture of *T.b. brucei* AnTaR 1, *T.b. rhodesiense* RUMPHI and the *T. b. gambiense* strains LiTaR 1 and 348 BT are described in Table 1. The propagation of the bloodstream form *in vivo* in rodents, the adaptation *in vitro* to an HMI-9 based culture medium and the molecular confirmation of their taxonomic identity have been described previously [7], [51], [52]. Iscove's modified Dulbecco's medium powder (IMDM) and foetal calf serum (FCS; heat-inactivated; EU approved; South American origin) were purchased from Invitrogen (Carlsbad, USA). For *in vitro* assays, medium was prepared from IMDM without phenol red (Invitrogen) and without addition of antibiotics. All other culture media ingredients were from Sigma–Aldrich (St. Louis, MO, USA). Briefly, strains were isolated from first peak parasitaemia in mice, cultured in HMI-9 based medium containing 1,1% methylcellulose and 15% foetal bovine serum with or without 5% heat-inactivated human serum until adaptation [51]. All strains were adapted to medium without methylcellulose before transfection as previously described [52]. Strains were cultivated in 500 µl of medium in a 48-well plate at densities between 10^3^–10^6^ cells ml^−1^ and maintained in logarithmic growth phase by subpassages at appropriate dilutions after 24 to 72 hours of incubation at 37°C and 5% CO_2_. Cultures were monitored by phase contrast inverted microscopy. Cell counting was performed in disposable counting chambers (Uriglass, Menarini Diagnostics, Belgium). For larger cell preparations, the cultures were stepwise scaled up to 40 ml in 25 cm^2^ flasks, by addition of four (for *T.b. gambiense*) to nine (for *T.b. rhodesiense/brucei*) volumes of fresh medium once the parasites reached a density of 5×10^5^ cells ml^−1^. For long term storage, cells were concentrated tenfold from log phase cultures in 90% medium with 10% glycerol and frozen stepwise to −40°C at 1°C/min using a programmable cryogenic freezing device (MiniCool MP40, Air Liquide, Belgium) whereafter they were kept in liquid nitrogen.

### Overexpression of reporter genes

The promoterless vector pHD309 was used for constitutive expression of foreign genes in trypanosomes [5], [30], [52]. For overexpression of a single reporter gene, the pHD309 plasmid was cut with BamHI and HindIII and PCR products were fused using In-Fusion Cloning and transformed in Fusion Blue cells according to the manufacturer's recommendations (Clontech, Takara Bio, Japan). The cDNA sequences of the reporter genes were amplified from their donor plasmids using gradient PCR and a proofreading polymerase (Deep Vent_R_, New England Biolabs, UK). All primers (Biolegio, Nijmegen, The Netherlands) contained a cDNA specific sequence and a 5′ extension of 15 nucleotides specific to the place of integration, containing the restriction site and sequence overlap with the vector as required for the In-Fusion Cloning reaction (Table S1). Trypanosomes that have been electroporated (Gene Pulse Xcell, Bio-Rad, USA) with a NotI linearised plasmid can afterwards be selected with hygromycin to obtain stable recombinants. The transfection and selection of trypanosome strains that express RLuc has been described earlier [30], [52]. Recombinant populations were maintained in 1 µg ml^−1^ hygromycin for *T.b. gambiense* strains and 5 µg ml^−1^ for *T.b. brucei* and *T.b. rhodesiense* strains for over three weeks after transfection upon which the most resistant populations were cryopreserved and used for further analysis of luciferase activity. The selection antibiotic was no longer added to the *in vitro* cultures during luciferase activity and drug sensitivity testing.

### *In vitro* luciferase activity

To measure the luminescent activity of the RLuc-modified strains, the EnduRen Live Cell assay (Promega, Madison, USA) was used with a final EnduRen concentration of 6 µM. Sixty mM EnduRen stock solution in DMSO was diluted 1∶1000 into HMI-9 medium without phenol red. Five µl of this solution were transferred to a well of a white opaque 1/2 area 96-well plate (Perkin Elmer, Waltham, MA, USA) and 45 µl of a trypanosome suspension were added. The plate was incubated for at least one hour in a 5% CO_2_ incubator at 37°C. After measurement of RLuc activity with EnduRen, the amount of ATP was measured in the same sample by adding 50 µl of CellTiter Glo reagent (Promega), to create a luminescent multiplex viability assay as described previously [52]. To measure the luminescent activity of the firefly luciferases, the ONE-Glo Luciferase reagent (Promega) was reconstituted as described by the manufacturer and 20 µl of this assay solution were added to 20 µl of a trypanosome suspension in HMI-9 in an opaque white 1/2 area 96-well plate (Perkin Elmer). A separate aliquot of 20 µl of the trypanosome suspension was used to measure ATP luminescence using the CellTiter-Glo reagent. No centrifugation or wash steps were required in any of the protocols. All luminescent measurements were performed after 2 minutes of shaking and the number of counts was integrated by sampling over a 1 second period (CPS), every minute for 10 minutes using WorkOut software from Victor X3 plate reader (Perkin Elmer). The CPS values were divided by the CPS value of HMI-9 medium (fold change), plotted against the trypanosome cell density and a linear regression was calculated in GraphPad (Prism). The threshold for detection was defined as a fold change >3. The relative activity in each clone was calculated as the ratio of the CPS in the luciferase assay (EnduRen, for RLuc or ONE-Glo, for CBR and P9) over the CPS in the luminescent cell viability assay (CellTiter Glo). The means of the clones of the red-shifted luciferases with the highest relative activity and the mean doubling time of the wild-type and their luminescent population(s) were compared using one-way analysis of variance (ANOVA) with Bonferroni *post-hoc* test in GraphPad (Prism).

### IC_50_ drug sensitivity

Eflornithine (Sanofi Aventis, Paris, France) and hygromycin B (Sigma) were prepared as 10 mg ml^−1^ stock solutions in distilled water. Melarsoprol (Sanofi Aventis), suramin (Bayer, Leverkusen, Germany), pentamidine isethionate (Sanofi Aventis) and nifurtimox (Sigma) were stored as 10 mg ml^−1^ stock solutions in DMSO. Dophanil powder (Docpharma, Hoeilaart, Belgium), containing 455 mg diminazene diaceturate and 555 mg antipyrine per gram, was prepared as a 10 mg ml^−1^ diminazene diaceturate solution in DMSO. A method to measure the IC_50_ values of compounds in 96-well plates was performed as described elsewhere [53]. Threefold drug dilutions in duplicate were made in HMI-9 medium to allow testing in final drug concentrations ranging from 100 to 0.14 µg ml^−1^ for eflornithine and hygromycin, from 50 to 0.07 µg ml^−1^ for nifurtimox, from 10 to 0.014 µg ml^−1^ suramin and from 500 to 0.7 ng ml^−1^ for diminazene diaceturate, melarsoprol and pentamidine with 5×10^3^ cells ml^−1^ in a total volume of 200 µl. Next, the plate was incubated for 72 hours at 37°C with 5% CO_2_ followed by addition of 20 µl of resazurin (Sigma; 12.5 mg in 100 ml PBS). After a further 24 h incubation at 37°C with 5% CO_2_, fluorescence was measured (excitation λ = 560 nm; emission λ = 590 nm) with a VictorX3 multimodal plate reader using top reading (Perkin Elmer) [54]. The results were expressed as the percent reduction in parasite viability compared to the parasite viability in control wells without drugs. The 50% inhibitory concentration (IC_50_) was calculated using non-linear regression and compared between groups with one-way ANOVA and Bonferroni *post-hoc* test in GraphPad (Prism).

### *In vivo* luciferase activity

**Figure 1:**
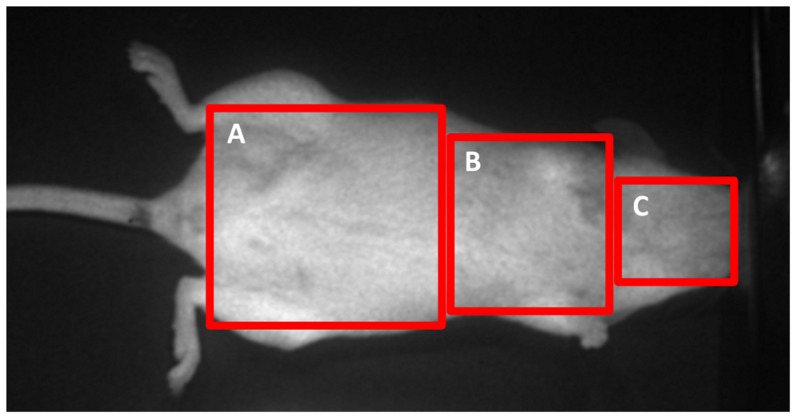
Definition of ROI. BLI data were analysed in function of 3 ROIs; (A) abdomen, (B) thorax and (C) head.

For experiments with *T.b. brucei* AnTaR 1, *T.b. gambiense* LiTaR 1 and *T.b. rhodesiense* RUMPHI, female OF-1 mice (25±3 g) in groups of 3 were infected intraperitoneally (IP) with 2×10^4^ parasites (from culture medium). Every group was tested at days 1, 4, 7, 18 and 26 post-infection. For experiments with *T.b. gambiense* 348 BT, female OF-1 mice (30±5 g) in groups of 3, treated or not treated with 200 mg/kg cyclophosphamide (CPA) IP (Endoxan, Baxter, Lessing, Belgium) 2 days pre-infection, were infected with 2×10^5^ parasites (from infected mouse blood). These groups were tested at days 1, 3, 7, 11, 43 and, for CBR only, also on day 60 post-infection. Before each BLI recording, animals were weighed and anaesthetised by inhalation of 5% isoflurane (Isoflo, USP) for induction and 2% isoflurane for maintenance in 100% 0_2_ at a flow rate of 1000 ml min^−1^. While under anaesthesia, mice were injected IP with 10 ml kg^−1^ body weight of a 15 mg ml^−1^ D-luciferin (ViviGlo, D-luciferin potassium salt, Promega) in phosphate buffered saline pH 7.4 (PBS) or 1 mg ml^−1^ ViviRen (Promega) in PBS with 0,1% bovine serum albumin (BSA) [55]. Two to five minutes after injection of the substrate, a ten to fifteen minute image acquisition was made on an *in vivo* bioluminescence imager (Photon Imager, Biospace, France). During the imaging session, the animal was placed on its back on a heated mat (39°C) to maintain body temperature. After each session, the parasitaemia was estimated using the matching method on 30 fields, allowing a detection limit of 10^5^ cells ml^−1^ in whole blood [56]. The BLI data were analysed by dividing the images of the mice in 3 rectangular shaped regions of interest (ROI); covering the abdomen (12.3 cm^2^), the thorax (6.1 cm^2^) and the head (2.9 cm^2^) (Figure 1). The radiance in each ROI was obtained from a 30 to 60 second period within the plateau phase of luminescence and expressed in photons per second per square centimetre per steradian (ph s^−1^ cm^−2^ sr^−1^) in M3 Vision (Biospace, France). In the non-infected controls ViviRen was injected first, followed by a washout period of at least 4 hours before D-luciferin administration. The threshold for detection was defined as a >3 fold change in radiance compared to non-infected controls.

### *Ex vivo* luciferase activity

At day 43 and day 60 for *T.b. gambiense* 348 BT and at day 26 for *T.b. brucei* AnTaR 1 and *T.b. rhodesiense* RUMPHI, mice were transcardially perfused, under Nembutal anaesthesia (60 mg kg^−1^ in PBS, IP), with 50 ml of phosphate buffered saline glucose (PBSG; 10 mM phosphate pH 7.4, 0.9% NaCl and 1% glucose) at a flow rate of 5 ml min^−1^ to rinse the vascular compartments of trypanosomes. The spleen was excised and weighed while the brains (without dura mater and arachnoid) were removed, washed in 10 ml of PBSG and incubated in a 24-well plate in 1 ml of PBSG containing either 1.5 mg ml^−1^ D-luciferin or 0,1 mg ml^−1^ ViviRen. After 5 minutes delay, a BLI recording was made for 10 minutes. The BLI data were analysed by drawing a ROI around the circumference of the well. The radiance (ph s^−1^ cm^−2^ sr^−1^) was expressed as fold change over the average values of the non-infected control brains for each substrate as described above.

## Results

### A panel of blue and red luminescent trypanosomes

**Figure 2:**
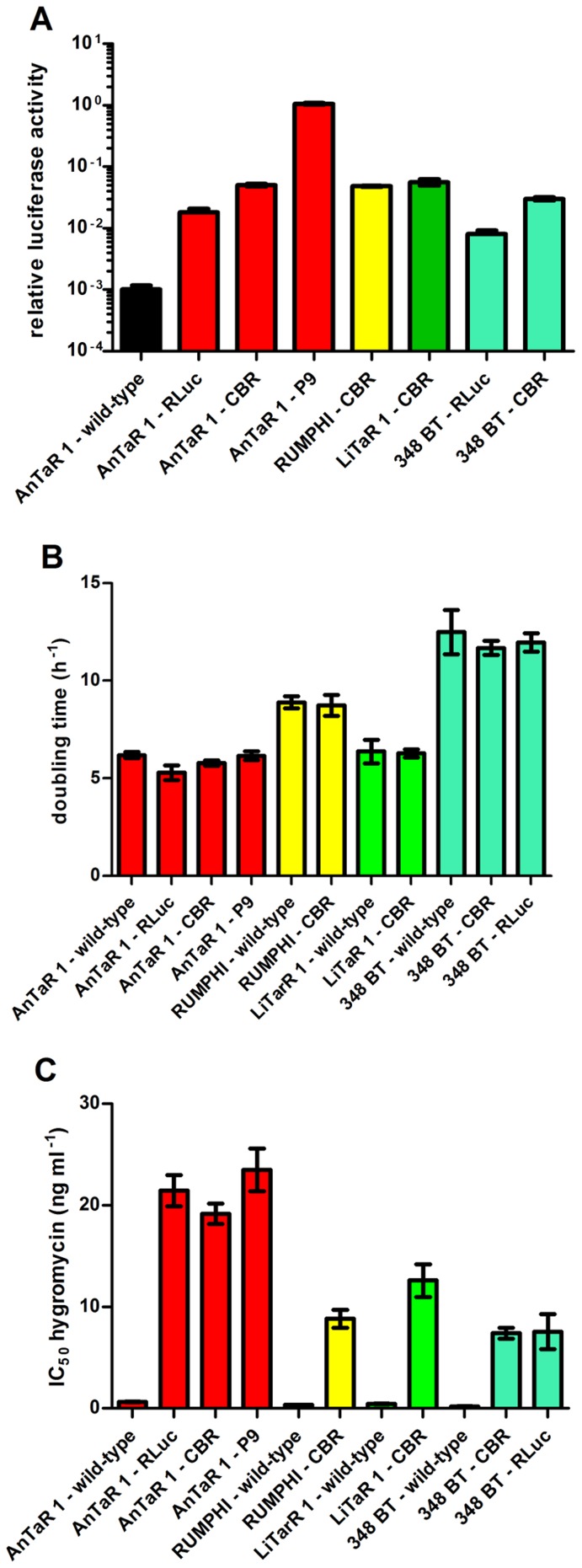
Characteristics of wild-type and luminescent strains. Comparison of (A) relative luciferase activity (values are the mean ± SD from 6 to 11 cultures) (B) doubling time (values are the mean ± SD from 3 cultures) and (C) hygromycin resistance IC_50_ (values are the mean ± SD from 2 to 7 cultures).

RLuc luciferase expressing clones of *T.b. brucei* AnTaR 1 and *T.b. gambiense* 348 BT were available from previous studies [30], [52]. CBR luciferase was integrated in these strains as well as in *T.b. gambiense* LiTaR 1 and *T.b. rhodesiense* RUMHPI. The PpyRE9 luciferase (P9), a promising red-shifted luciferase for *in vivo* imaging, was only integrated in *T.b. brucei* AnTaR 1 and was not yet tested in the other strains. Out of four clones of *T.b. brucei* AnTaR 1 transfected with pHD P9 (AnTaR 1 P9), ten clones of *T.b. brucei* AnTaR 1 transfected with pHD CBR (AnTaR 1 CBR), 3 clones of *T.b. rhodesiense* RUMPHI transfected with pHD CBR (RUMPHI CBR), 7 clones of *T.b. gambiense* LiTaR 1 transfected with pHD CBR (LiTaR 1 CBR) and 2 clones of *T.b. gambiense* 348 BT transfected with pHD CBR (348 BT CBR) that were simultaneously tested, we identified for each strain and luciferase reporter combination, the clone with the highest relative luciferase activity (Figure 2: A and Figure S1). There was a significant difference in relative luciferase activity among these red luminescent clones (ANOVA, F(5,39) = 751; *p*<0.0001). *Post-hoc* analysis revealed that the relative luciferase activity of clone 4 of P9-modified *T.b. brucei* AnTaR 1 was significantly higher than the highest relative luciferase activity of the CBR-modified clones from *T.b. brucei* AnTaR 1 (clone 19), *T.b. gambiense* LiTaR 1 (clone 14), *T.b. rhodesiense* RUMPHI (clone 51) and *T.b. gambiense* 348 BT (clone 13) (*p*<0.05). When comparing only the CBR-tagged strains (ANOVA, F(4,32) = 48.5; *p*<0.0001), *post-hoc* analysis showed that the relative luciferase activity of *T.b. gambiense* 348 BT CBR was significantly lower than the others (*p*<0.05). We found that at least 10^5^ cells ml^−1^ were necessary for detection of the most luminescent clones among the CBR-modified strains, while for *T.b. brucei* AnTaR 1 P9 5×10^3^ cells ml^−1^ of clone 4 were sufficient (Figure S2). In the luminescent viability assay, at least 1×10^4^ cells ml^−1^ were needed to obtain a >3 fold change for all tested strains (Figure S3).

The *in vitro* growth rates (expressed as doubling time) were significantly different between the collection of strains (ANOVA F(10,22) = 30.9; *p*<0.0001). However, *post-hoc* analysis revealed no significantly different growth rates of the wild-type and the corresponding recombinant population(s) of each strain (*p*>0.05) (Figure 2: B). The IC_50_ values of the luminescent populations for hygromycin were significantly higher than those of the corresponding wild type populations (ANOVA F(10,49) = 46.98, p<0.0001). *Post –hoc* analysis revealed no difference in IC_50_ values for hygromycin between the luminescent *T.b. brucei* AnTaR 1 clones (p>0.05), but the IC_50_ values of *T.b. gambiense* LiTaR 1 CBR, *T.b. rhodesiense* RUMPHI CBR and both CBR- and RLuc-modified *T.b. gambiense* 348 BT strains were significantly lower than those of the luminescent *T.b. brucei* AnTaR 1 populations (*p*<0.05) (Figure 2: C).

### IC_50_ values of the wild-type and recombinant strains for trypanocides

**Figure 3:**
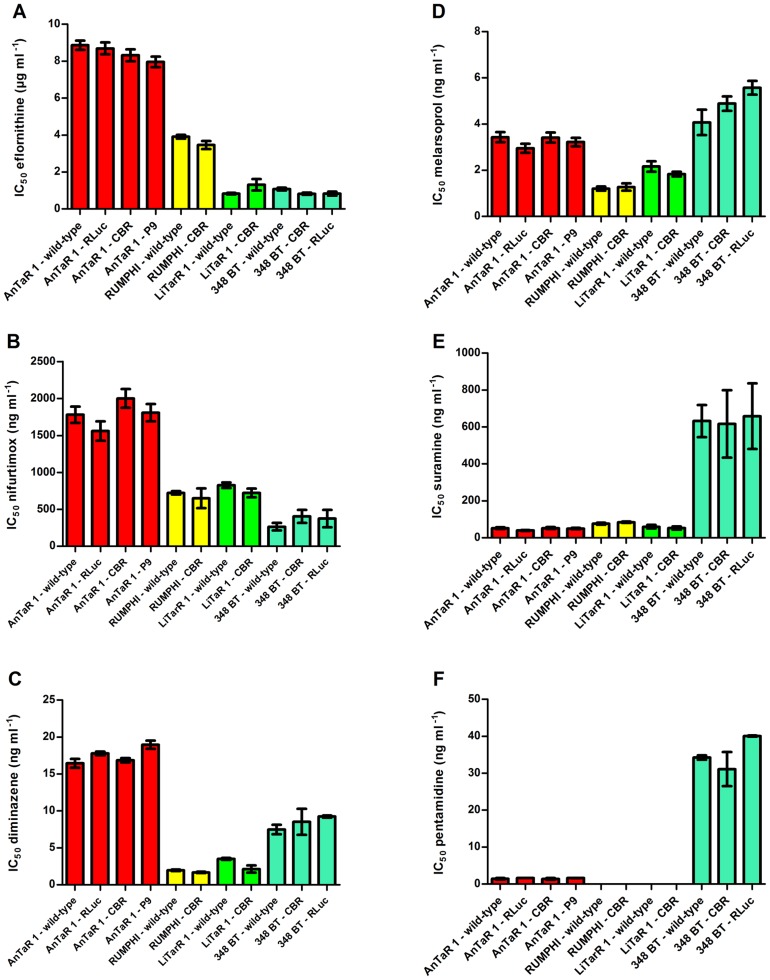
Drug sensitivity profiles of wild-type and luminescent strains. IC_50_ (mean ± SD) of wild-type and luminescent strains against (A) eflornithine (values are the mean ± SD from 4 to 9 cultures), (B) nifurtimox (values are the mean ± SD from 4 to 9 cultures), (C) diminazene diaceturate (values are the mean ± SD from 2 cultures), (D) melarsoprol (values are the mean ± SD from 4 to 9 cultures), (E) suramin (values are the mean ± SD from 4 to 9 cultures) and (F) pentamidine isethionate (values are the mean ± SD from 2 cultures).

The drug sensitivity profiles of all wild-type and luminescent strains were compared against a set of trypanocides (eflornithine, nifurtimox, diminazene diaceturate, melarsoprol, suramin and pentamidine isethionate) to test if the luminescent modifications induced differences in IC_50_ value. For each drug, ANOVA found differences between the IC_50_ values from the collection of strains (for eflornithine F(10,66) = 183, *p*<0.0001; for nifurtimox F(10,66) = 37, *p*<0.0001; for diminazene diaceturate F(10,11) = 112.3, *p*<0.0001; for melarsoprol F(10,63) = 33, *p*<0.0001; for suramin F(10,68) = 26.22, *p*<0.001 and for pentamidine F(6,7) = 105, p<0.0001). *Post-hoc* analysis did not reveal significant differences between the IC_50_ values of the wild-type and the corresponding luminescent population(s) of each strain (for each drug, *p*>0.05). However, there were substantial differences between the different strains for each of the different drugs as represented in Figure 3. For eflornithine, all *T.b. brucei* AnTaR 1 populations had significantly higher IC_50_ values than all the other populations and all *T.b. rhodesiense* RUMPHI populations had significantly higher IC_50_ values than *T.b. gambiense* populations (*p*<0.05) (Figure 3: A). For nifurtimox, the IC_50_ values of all *T.b. brucei* AnTaR 1 populations were significantly higher, while all IC_50_ values of *T.b. gambiense* 348 BT were significantly lower than all IC_50_ values of *T.b. rhodesiense* RUMPHI and *T.b. gambiense* LiTaR 1 populations (*p*<0.05) (Figure 3: B). For diminazene diaceturate, IC_50_ values of all *T.b. brucei* AnTaR 1 and *T.b. gambiense* 348BT were significantly different and were significantly higher IC_50_ than of all *T.b. rhodesiense* RUMPHI and *T.b. gambiense* LiTaR 1 populations (*p*<0.05) (Figure 3: C). For melarsoprol, IC_50_ values of all *T.b. brucei* AnTaR 1 and *T.b. gambiense* 348 BT populations were higher than those of *T.b. gambiense* LiTaR 1 and *T.b. rhodesiense* RUMPHI (Figure 3: D). Strikingly, the IC_50_ values of all *T.b. gambiense* 348 BT for suramin were about tenfold higher than of all the other strains (*p*<0.05) (Figure 3: E). Also for pentamidine, the IC_50_ values of all *T.b. gambiense* 348 BT populations were much higher than those of *T.b. brucei* AnTaR 1, while the IC_50_ values of *T.b. gambiense* LiTaR and *T.b. rhodesiense* RUMPHI were below the lower threshold (<0.70 ng ml^−1^) (*p*<0.05)(Figure 3: F).

### Infections with the different luciferase-tagged *T.b. brucei* AnTaR 1 trypanosomes

The sensitivity of RLuc, CBR and PpyRE9 luciferase detection during *in vivo* BLI of a murine infection was assessed with *T.b. brucei* AnTaR 1. Throughout the infection, very high parasitemia between 10^7^ and 10^8.4^ cells ml^−1^ was observed for the wild-type strain and for the RLuc-, CBR- and P9-modified clones. Mice infected with wild-type and recombinant *T.b. brucei* AnTaR 1 parasites showed increased body weight gain. The spleen weight of *T.b. brucei* AnTaR 1 infected mice that survived until day 26 post-infection varied from 1 to 2 gram, roughly tenfold the spleen weight of uninfected mice, indicating severe splenomegaly.

**Figure 4:**
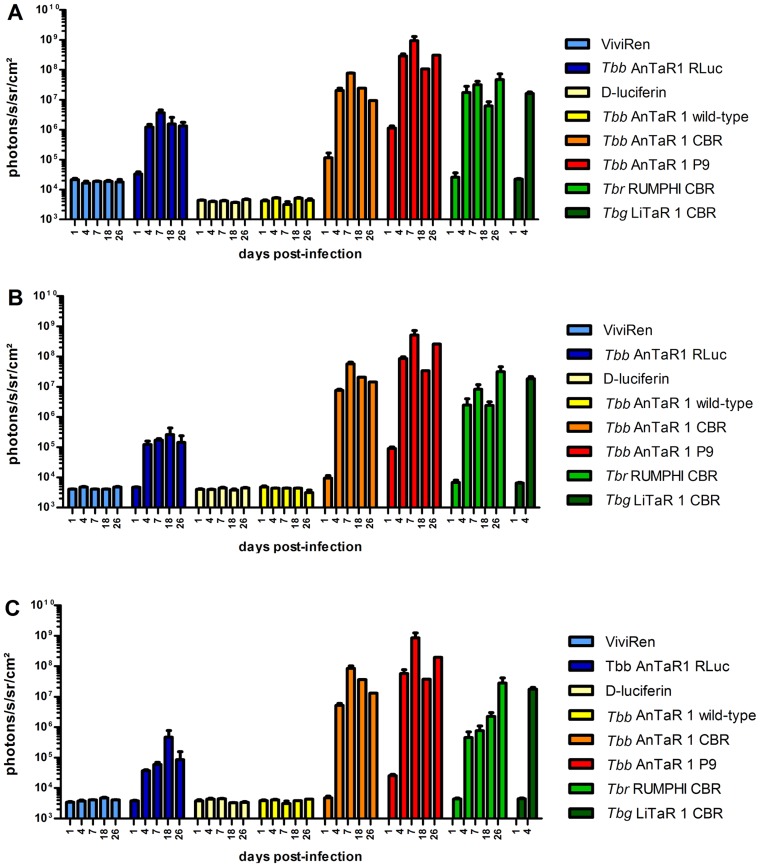
Quantification of BLI data of OF-1 mice infected with luminescent *T.b. brucei*, *T.b. rhodesiense* and *T.b. gambiense*. OF-1 mice (n = 3) were infected with *T.b. brucei* AnTaR 1 wild-type, *T.b. brucei* AnTaR 1 RLuc, *T.b. brucei* AnTaR 1 CBR, *T.b. brucei* AnTaR 1 P9, *T.b. rhodesiense* RUMPHI CBR, *T.b. gambiense* LiTaR 1 CBR and their luminescence was measured at 1, 4, 7, 18 and 26 post-infection in BLI using their respective substrates. The BLI data were divided in 3 ROIs; (A) abdomen, (B) thorax and (C) head and expressed as ph s^−1^ cm^−2^ sr^−1^.

**Figure 5:**
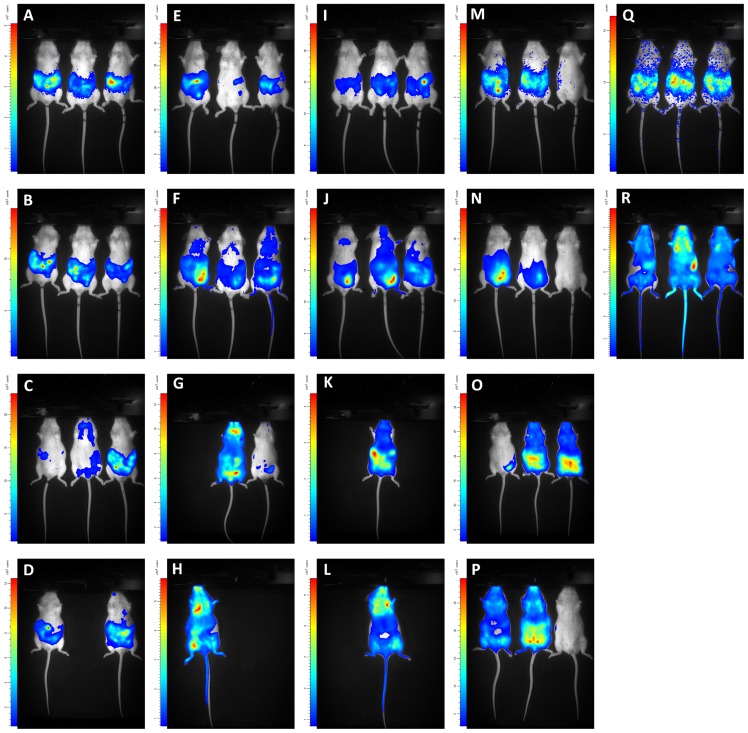
Visualisation of BLI data of OF-1 mice infected with *T.b. brucei*, *T.b. rhodesiense* and *T.b. gambiense*. OF-1 mice (n = 3) were infected with *T.b. brucei* AnTaR 1 RLuc (A–D), *T.b. brucei* AnTaR 1 CBR (E–H), *T.b. brucei* AnTaR 1 P9 (I–L), *T.b. rhodesiense* RUMPHI CBR (M–P), *T.b. gambiense* LiTaR 1 CBR (Q–R) and their luminescence was measured in BLI using their respective substrates. Rows represent measurements at 1, 4, 18 and 26 days post-infection.

For the BLI experiments, the background luminescence of each luciferase substrate was measured in uninfected mice and was at least four times higher for ViviRen (16228±4826 ph s^−1^ cm^−2^ sr^−1^) than for D-luciferin (4622±927 ph s^−1^ cm^−2^ sr^−1^) in the abdominal ROI, with lesser differences in the thoracic ROI (ViviRen: 4064±873 and D-luciferin: 3743±927 ph s^−1^ cm^−2^ sr^−1^) and in the head ROI (ViviRen :3772±789 and D-luciferin: 3131±459 ph s^−1^ cm^−2^ sr^−1^). The *in vivo* luciferase activities in function of ROI and days post-infection are represented in Figure 4 and visualised in Figure 5. At day 1 post-infection, when parasitaemia is not yet detectable by microscopy, the BLI signal is already above threshold in the abdominal region of some mice infected with the CBR strain (Figure 5: E) and in all compartments of all mice infected with the P9 strain (Figure 5: I). No signal above background could be detected in mice infected with RLuc-tagged parasites (Figure 5: A). At day 4 post-infection, the RLuc-infected mice (Figure 5: B) were 100 fold more luminescent in the abdominal region and at least 10 fold more luminescent in the thorax and the head region than wild-type-infected mice (Figure 4). Mice infected with trypanosomes expressing red luciferases were much more luminescent with a fold change of 5000 in the abdomen and 1000 in the thorax and head for CBR-infected mice and a fold change of almost 100000 in the abdomen and of 10000 in the thorax and head for the P9-infected mice (Figure 4 and Figure 5: F and J).

Interestingly, the difference in sensitivity (as defined by higher radiance values) between CBR and P9 was not associated with a different distribution of the trypanosomes in the body. No more visual detail could be obtained from the higher luminescence of P9 compared to CBR-tagged parasites (Figure 5: F and J, G and K). BLI pictures often revealed the contours of lymph nodes, but in most cases the signal consisted of a superimposed surface covering multiple organs in the body, especially in the abdomen. With RLuc-tagged parasites, less information was obtained. Luminescence was often only visible in the abdominal region (Figure 5: B, C and D). After day 7 post-infection, data were too limited for analysis due to differences between the strains in survival of mice and parasitaemia. The median survival time was 26 days for mice infected with wild-type; 26 days with RLuc; 26 days with CBR and 11 days with P9, with deaths in all groups occurring first at day 11 post-infection (3 animals) and later between day 18 and 25 post-infection (4 animals).

### Infections with CBR-tagged trypanosomes of the different *T.b.* subspecies

*T.b. gambiense* LiTaR 1 killed mice in 5 days without any overt signs of pathology. The trypanosomes were monomorphic and at day 4 post-infection, hours before death, the parasitaemia approached 10^8,7^ cells ml^−1^. In contrast, *T.b. rhodesiense* RUMPHI infection was not fatal up to day 26 post-infection, which marked the end of the experiment. Throughout the infection, the parasitaemia of *T.b. rhodesiense* RUMPHI varied between 2×10^6^ and 5×10^7^ cells ml^−1^ and was markedly lower than that of *T.b. brucei* AnTaR 1 and *T.b. gambiense* LiTaR 1. One mouse showed a delay in reaching the first peak of parasitemia (Figure 5: M–O). Although mice infected with RUMPHI did show signs of lethargy, splenomegaly was less pronounced than in mice infected with *T.b. brucei* AnTaR 1. When we compared the *in vivo* luciferase activity in mice infected with CBR-tagged *T.b. brucei* AnTaR 1, *T.b. rhodesiense* RUMPHI and *T.b. gambiense* LiTaR 1, it appeared that on day 1 post-infection, when none of the infected mice showed microscopically detectable parasitaemia, all strains could be detected with BLI in the abdominal region of some infected mice (Figure 5: E, M and Q). At day 4 post-infection, all three strains generated a comparable BLI signal in the abdomen and thorax (Figure 4: A and B). The signal from the head was highest in mice infected with *T.b. gambiense* LiTaR 1, followed by mice infected with *T.b. brucei* AnTaR 1 and with *T.b. rhodesiense* RUMPHI (Figure 4: C and Figure 5: F, N and R). Later during the infection, all mice infected with *T.b. gambiense* LiTaR 1 and 2 of 3 mice infected with *T.b. brucei* AnTaR 1 died while all mice infected with *T.b. rhodesiense* RUMPHI survived until the end of the experiment at day 26 post-infection with steadily increasing BLI signals in the head ROI (Figure 5: O and P).

### Effect of immune suppression on infection with blue and red *T.b. gambiense* 348 BT

**Figure 6:**
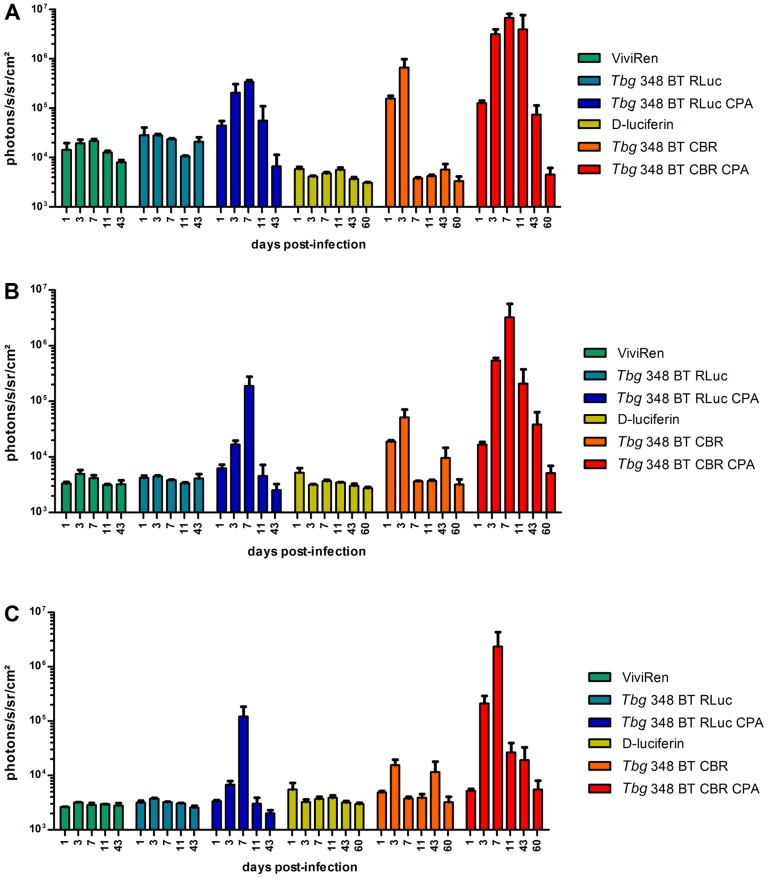
Quantification of BLI data in untreated and CPA-treated mice infected with *T.b. gambiense*. OF-1 mice (n = 3) untreated or CPA-treated were infected with *T.b. gambiense* 348 BT RLuc and *T.b. gambiense* 348 BT CBR and their luminescence was measured at 1, 3, 7, 11, 43 (for RLuc) and 60 days post-infection (for CBR) using their respective substrates. The BLI data were divided in 3 ROIs; (A) abdomen, (B) thorax and (C) head and expressed as ph s^−1^ cm^−2^ sr^−1^.

**Figure 7:**
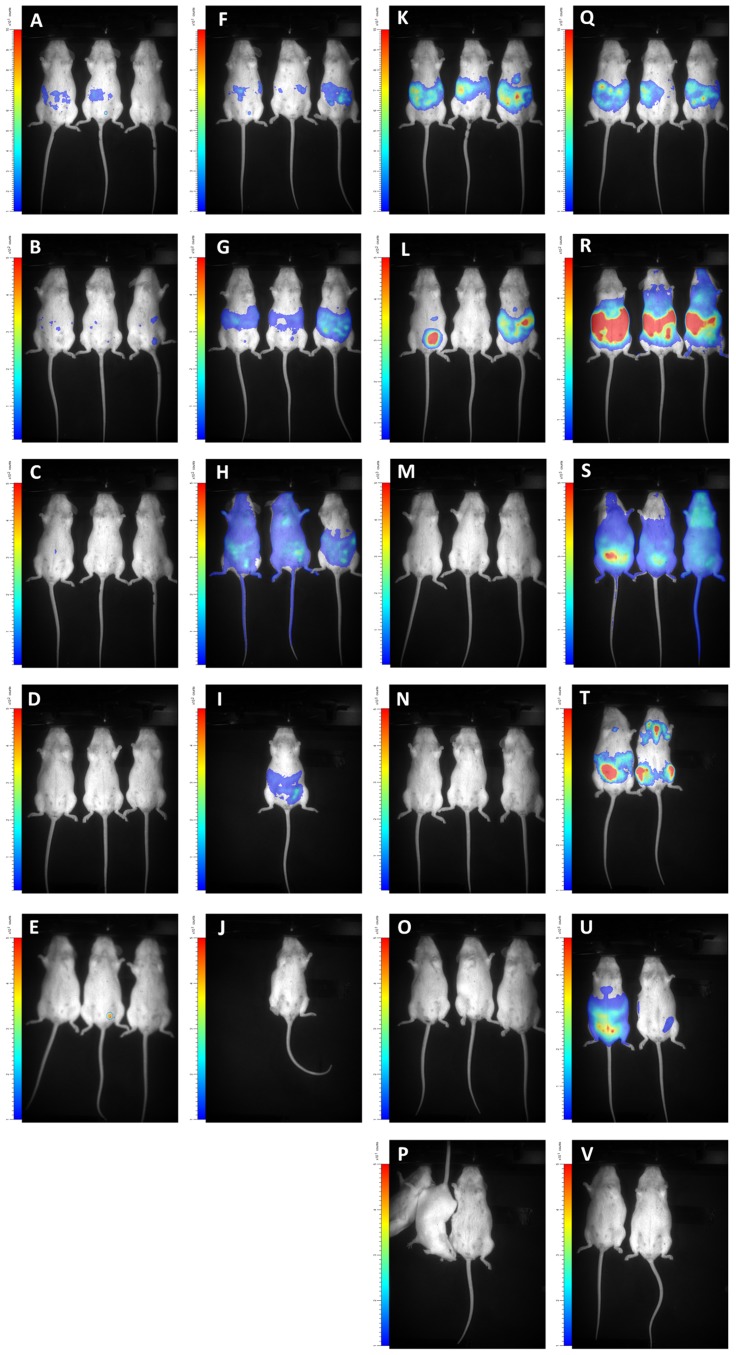
Visualisation of BLI data in untreated and CPA-treated OF-1 mice infected with *T.b. gambiense*. OF-1 mice (n = 3) were infected with *T.b. gambiense* 348 BT RLuc without (A–E) or with CPA treatment (F–J) and their luminescence was measured with ViviRen or were infected with *T.b. gambiense* 348 BT CBR without (K–P) or with (Q–V) CPA treatment and their luminescence was measured with D-luciferin. Rows represent measurements at 1, 3, 7, 11, 43 (for RLuc) and 60 days post-infection (only CBR). In square P one mouse woke up from anesthesia just before the end of acquisition.

RLuc- and CBR-modified parasites of *T.b. gambiense* 348 BT were injected in BALB/c that underwent weekly CPA treatment. When these animals became parasitologically positive after 14 to 20 days (a total of 2–5 trypanosomes in 30 fields), their blood was injected into CPA-treated OF-1 mice. The peak of parasitaemia occurred within one week and again, blood containing the parasites was injected in 2 CPA-treated OF-1 mice. The first peak of parasitaemia in the latter mice reached 10^8,1^ cells ml^−1^ and blood was diluted with PBSG to 10^6^ cells ml^−1^ and used to infect three CPA-treated OF-1 mice and three untreated OF-1 mice. In the untreated mice, parasitaemia remained undetectable in all RLuc-infected mice and in two CBR-infected mice; only one CBR-infected mouse was once positive at day 4 post-infection. In the CPA-treated mice, both RLuc and CBR infections gave rise to detectable parasitaemia that increased until 11 days post-infection where after the mice became only sporadically positive. At the end of the experiment we recorded mild splenomegaly in all mice that became parasitologically positive. The *in vivo* luciferase activity in function of ROI and days post infection are represented in Figure 6 and visualised in Figure 7. As observed in the BLI experiment with *T.b. brucei* AnTaR 1, the *T.b. gambiense* 348 BT RLuc-modified parasites were less informative than the CBR-modified parasites. With BLI, the signal of the RLuc-modified trypanosomes was below threshold during the whole infection period in untreated mice (Figure 7: A–E), but in CPA-treated animals, the *T.b. gambiense* 348 BT RLuc-modified parasites were detectable at day 1 and day 3 post-infection in the abdomen (Figure 7: F and G) and at day 7 also in the thorax and the head of some mice (Figure 7: H). In contrast, in both CPA-treated and untreated mice, *T.b. gambiense* 348 BT CBR-tagged parasites were detectable at day 1 post-infection in the abdomen, and at day 4 post-infection also in the thorax and the head (Figure 7: K–V). At day 7 post-infection, the *T.b. gambiense* 348 BT CBR-modified parasites became undetectable in the CPA-untreated mice (Figure 7: M–P). In the CPA-treated mice we were able to track the infection in all animals and in all compartments until day 43 post-infection, yet at day 60 post-infection the signal decreased below the detection threshold (Figure 7: V).

### *Ex vivo* luminescence in the brain

**Figure 8:**
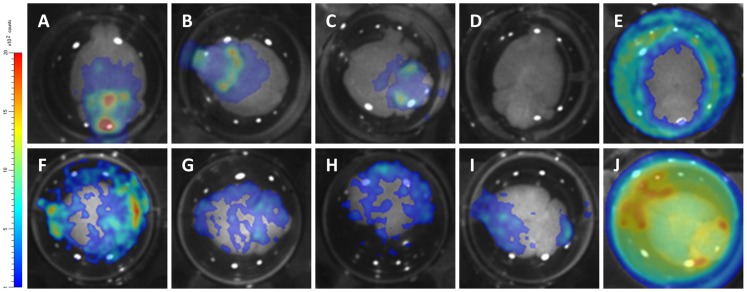
Visualisation of *ex vivo* brain BLI data obtained from mice infected with different luminescent strains. At 26 days post-infection for *T.b. brucei* AnTaR 1 and *T.b. rhodesiense* RUMPHI and at 43 and 60 days post-infection for *T.b. gambiense* 348 BT brains were extracted and immersed in PBSG and substrate. (A–B) *T.b. gambiense* 348 BT CBR, CPA-treated, with D-luciferin; (C) *T.b. gambiense* 348 BT CBR, untreated, with D-luciferin ;(D) uninfected with D-luciferin; (E) *T.b. gambiense* 348 BT RLuc, CPA-treated with ViviRen; (F–H) *T.b. rhodesiense* RUMPHI CBR with D-luciferin; (I) *T.b. brucei* AnTaR 1 CBR with D-luciferin; (J) *T.b. brucei* AnTaR 1 P9 with D-luciferin.

At day 26 post-infection for *T.b. brucei* AnTaR 1 and *T.b. rhodesiense* RUMPHI and at day 43 and day 60 post-infection for *T.b. gambiense* 348 BT, the surviving animals were sacrificed for *ex vivo* BLI quantification of the parasites in the brain. The ventral portion of the brain was the most informative for BLI data (Figure 8). The brains from the animals infected with CBR-tagged strains (Figure 8: A–C, I) showed 50 to 100 fold higher luminescence than brains of uninfected mice (Figure 8: D, representative image measured with D-luciferin). In the brain of the mouse infected with the *T.b. brucei* P9-tagged strain (Figure 8: J), the luminescence was even about 1000 fold higher. In the case of infection with *T.b. gambiense* 348 RLuc and *T.b. brucei* AnTaR RLuc, luminescence was detectable at the circumference of the brain rather than in the brain itself (Figure 8: E, image of *T.b. gambiense* 348 BT RLuc measured with ViviRen) and similar to the recording made from the brain of the *T.b. brucei* AnTaR P9 infected mouse (Figure 8: J), light radiated into the PBSG medium. However, we did not check whether we could detect free trypanosomes in the surrounding liquid or on the dorsal portion of the brain. In case of infections with CBR and P9 tagged trypanosomes and with D-luciferin as substrate, BLI signals emanated from all over the brain, but the densest spots were often observed in the olfactory bulbs, in the ventral anterior hypothalamic region including the suprachiasmatic nucleus, and in the cerebellum, as well as in the pituitary gland. The brains of two CPA-untreated animals infected with the *T.b. gambiense* 348 BT CBR as well as all untreated mice infected with *T.b. gambiense* 348 BT RLuc remained negative in BLI. As stated above, these animals also remained aparasitaemic in blood.

## Discussion

The aim of the present study was to assemble a set of genetically and phenotypically diverse bioluminescent *T.b.* strains and to assess the optimal reporter system (enzyme and substrate) for *in vivo* bioluminescent imaging of murine infections.

### Are red-shifted luciferases more advantageous to monitor tagged trypanosomes *in vivo*?

In previous studies, we used RLuc as the reporter gene in bioluminescent models of *T.b.* [5], [30], [52]. For the current study, we opted to replace RLuc for *in vivo* imaging by a red-shifted firefly luciferase reporter for several reasons. Coelenterazine, used for RLuc activity detection, has rather unfavourable kinetics *in vivo*. For example, it is thought not to pass the blood-brain barrier due to the abundance of P-glycoprotein pumps in brain vascular endothelium, whose efflux activity restricts access of coelenterazine to the parenchyma unless there is severe dysfunction of the blood-brain barrier [57]. Although this might be interesting for studying advanced neurological trypanosomiasis models, it does not reflect the precise timing of CNS infection by the parasite since trypanosomes have been observed in CNS before tight junctions are disrupted [58]. Novel variants of coelenterazine, such as ViViRen, are more resistant against auto-oxidation in serum, but similarly to coelenterazine, the eventual BLI signal highly depends on the route of their administration [59]. Furthermore, all coelenterazine variants are more expensive than D-luciferin. Also, in contrast to coelenterazine variants, the distribution of D-luciferin *in vivo* is fairly well characterised and optimised protocols for administration and anaesthesia are available [60]. Therefore, we did not extend our research into red-shifted *Renilla* luciferases that have been described recently, neither did we compare different administration routes [61]. We prefer IP injection which is the most practical and appropriate for D-luciferin, the substrate of all firefly and beetle luciferases, including the red-shifted variants such as CBR and PpyRE9 [37].

When reporter genes are to be compared, one should use the same vector background and the same assay to standardise expression and activity measurement [62]. We expressed the different reporter genes, RLuc, CBR and PpyRE9 in the same trypanosomal expression vector, pHD309. This vector can be integrated in the β-tubulin locus of trypanosomes and is one of the few known expression vectors that has been proven successful in *T.b. gambiense* through simple electroporation. Genome data show that this locus consists of multiple tandem repeats, thus allowing multiple integrations, while among different *T.b.* strains, a wide variation in β-tubulin copy numbers has been reported [63], [64]. In our strains we did not assess the number of β-tubulin copy numbers or the number of reporter gene copies integrated in this locus but rather used hygromycin resistance to select modified clones of the same strain with equal IC_50_ values for comparison. Using this approach, our study confirms the higher catalytic activity of P9 compared to CBR in *T.b. brucei* AnTaR 1, both *in vitro* and *in vivo* with 10 to 20-fold higher signals generated by P9 as previously described by Branchini *et al* [37]. However, it should be noted that higher expression of reporter genes in *T.b.* can also be obtained by modifying the expression vector for RNA polymerase I dependent transcription and by replacing the 5′ and 3′ untranslated regions flanking the reporter gene with sequences that flank highly expressed genes, as demonstrated in McLatchie et al [40].

One of the limitations of this study is that we did not correlate the *in vivo* BLI data with precise quantification of the parasites, e.g. by real-time RT-PCR. However, we microscopically estimated parasitaemia in blood to allow comparison between luminescent clones of the same strain early in infection (day 4 to day 7). Furthermore, for CBR-modified *T.b. brucei* AnTaR 1, *T.b. rhodesiense* RUMPHI and *T.b. gambiense* LiTaR 1, clones were selected with the same level of luciferase activity per living cell, making differences in the *in vivo* BLI responses dependent on parasitaemia and distribution in organs. However, we did not verify whether the expression of the reporter genes remained similar during the infection. At least *in vitro* testing did not reveal changes in hygromycin resistance or luciferase activity during a 6 week period in culture.

With an inoculum of 2×10^4^ to 2×10^5^ parasites, the red luciferase trypanosomes can be tracked from day 1 post-infection, although the sensitivity of detection was higher with P9 than with CBR. This higher sensitivity of P9 is important to confirm infection prior to treatment where treatment is to be given very early after infection. Later in the infection, the added detail gained from higher sensitivity of P9 becomes less important since the low background signal inherent to *in vivo* luminescence imaging allows similar localisation of the trypanosomes within mice infected with CBR-modified trypanosomes, as previously reported by Close et al for FLuc and Lux comparison [65]. Compared to P9 and CBR, signals from RLuc modified trypanosomes are mainly from the abdominal region, which can be explained by the poor absorbance of ViviRen after IP administration. In contrast with their poor performance *in vivo*, protected coelenterazine compounds, like EnduRen (Promega), perform very well *in vitro* as was demonstrated in a luminescent multiplexed viability assay developed to monitor the response of RLuc-modified trypanosomes for compound screening [52].

### Is our collection of modified trypanosomes phenotypically diverse?

Our collection of luminescent trypanosomes contains strains with very diverse infection outcome. Although less virulent than *T.b. gambiense* LiTaR 1, *T.b. brucei* AnTaR 1 strains induce a subacute infection. The sustained high parasitaemia in the mice might take its toll on the cardiovascular, hepatic or splenic physiology causing some animals to die early in infection. The occurrence of hepatosplenomegaly is an indication of high virulence in *T.b. brucei* and *T. evansi* strains [66], [67]. Other studies record a less virulent phenotype of *T.b. brucei* AnTaR 1 that may be related to the number of passages in mice or *in vitro* or to the rodent species or breed. Our data suggest that the integration of the expression plasmid did not change the growth phenotype, as reflected by the *in vitro* doubling times of the wild-type and the recombinant strains. Because of this high lethality in mice, it would be advantageous to use rats as host, which are known to control the infection longer than mice [68], [69]. Unfortunately, *in vivo* imaging on larger rodents does not yet seem very efficient [70].

The *T.b. gambiense* LiTaR 1 strain induces the same high parasitaemia levels and early lethality as the monomorphic *T.b. brucei* Lister 427 strain [30]. In our *in vivo* experiments, the mice did not survive the first peak of parasitaemia. The *T.b. gambiense* LiTaR 1 has undergone numerous passages *in vivo* and has become monomorphic and highly virulent and thus very different from wild-type strains that cause chronic *gambiense* sleeping sickness. The investigation of *T.b. gambiense* LiTaR 1 is important since laboratory accidents have shown that this strains is very virulent also in humans [71].

*T.b. rhodesiens*e RUMPHI induces lower peak parasitaemia, does not show the survival bottleneck that characterises *T.b. brucei* AnTaR 1, but still, survival is limited to approximately one month. This strain is fully tsetse transmissible in *G. morsitans morsitans* (personal communication; Dr. Jan Van den Abbeele) and under pressure of normal human serum *in vitro*, the serum resistance associated (SRA) protein expressing phenotype can easily be obtained (data not shown). All experiments in the present study were performed without normal human serum pressure. It is not known if SRA expression would alter the virulence phenotype in the rodent model.

In contrast to the other strains in our luminescent collection, *T.b. gambiense* 348 BT mimics very well the chronic phenotype of classical *gambiense* sleeping sickness in humans. Although both RLuc and CBR modified parasites were visible in BLI early after infection of immunosuppressed mice, the BLI signal strongly decreased later during the infection reflecting progression to a chronic infection. Such chronic infections, similarly to what is observed in humans, are characterised by the absence of detectable parasites in blood but by presence of the parasites in the CNS. With *T.b. brucei*, such chronic infections with parasites only present in the CNS can only be obtained by subcurative treatment of the mice [17]. In our *in vivo* experiments, some animals infected with luminescent *T.b. gambiense* 348 BT remained negative in BLI. This may correspond to the silent infection phenotype of *T.b. gambiense*, in which trypanosomes are present in very low numbers and can only be traced back by histopathological or molecular assays [5]. In addition, our experiments show that with the same parasite strain a variety of infection phenotypes can be encountered in different individuals from the same outbred mouse strain, *in casu* OF-1 [20], [21].

With all pleomorphic parasite strains, the CNS infection was confirmed *ex vivo*. The sites that were found infected are not different from those previously described with other *T.b. gambiense* and *T.b. brucei* strains [5], [29], [41], [72]–[74]. It should be noted that we cannot report on the presence of the trypanosomes in the dura mater or the subarchnoid space because this tissue was removed during brain extraction. Furthermore, our sample size and time of brain extraction did not allow description of differences in CNS invasion between the different trypanosome strains.

Next to their diversity in virulence phenotype, the bioluminescent trypanosome strains display a wide diversity in drug sensitivity phenotype. *T.b. brucei* AnTaR 1 is less sensitive to eflornithine, nifurtimox and diminazene diaceturate than the human infective strains. Among the latter, *T.b. gambiense* 348 BT shows a particularly interesting phenotype. Compared to the other strains, it is less sensitive to melarsoprol, pentamidine and, surprisingly, to suramin. This strain originates from the HAT focus of Mbuji-Mayi in East Kasai, DRC where melarsoprol treatment failure rates of about 40% have been reported [47]. Other strains isolated from the same HAT focus have been found to be cross-resistant to melarsoprol and pentamidine, a phenotype that was linked to a chimaeric aquaglyceroporin 2/3 gene in their genome [48]. Sequencing confirmed the presence of the same mutation in *T.b. gambiense* 348 BT (personal communication, Dr. Mäser Pascal). A lower susceptibility of *T.b. gambiense* than *T.b. brucei* to suramin has been described previously, but an even higher resistance has been described in *T. evansi* [53], [54]. *T.b. rhodesiense* RUMPHI is less sensitive to eflornithine than the two *T.b. gambiense* strains in our collection but is fully sensitive to pentamidine. This finding is consistent with recent evidence that first stage *rhodesiense* HAT patients can be cured with pentamidine, a drug that is much less toxic than suramin [75], [76]. Yet, suramin is still the first line treatment for first stage *rhodesiense* HAT [1].

### Concluding remarks

We established a very diverse collection of bioluminescent *T.b. brucei*, *T.b. gambiense* and *T.b. rhodesiense* strains that is now available for *in vitro* and *in vivo* studies on trypanosomiasis research. For *in vivo* monitoring of murine infections, we recommend the use of trypanosome strains transfected with red-shifted luciferases, such as PpyRE9, above the blue RLuc. Recently it was shown that PpyRE9 was also advantageous to monitor disease progression with *T.b. brucei* GVR35. However, the study of *T.b. gambiense* field strains is far more relevant for sleeping sickness rodent models, because this parasite subspecies causes more than 95% of the cases of HAT and severely differs in virulence from *T.b. brucei*, causing more chronic and protracted infections in murine models. Furthermore, we modified a *T.b. gambiense* strain that harbours the AQP2/3 chimaeric gene, which was recently shown to be responsible for pentamidine and melarsoprol cross-resistance in field isolates and quite possibly contributes to the high treatment failure rates seen in the DRC and Southern Sudan.

## Supporting Information

Figure S1 Relative luciferase activity of wild-type and red-shifted luciferase modified trypanosomes. Relative luciferase activity (mean of 2–8 repetitions ± SD) of the firefly luciferase modified clones of several strains (*T.b. brucei* AnTaR 1, *T.b. rhodesiense* RUMPHI, *T.b. gambiense* LiTaR 1 and *T.b. gambiense* 348 BT).
(TIF)

Figure S2 ONE-Glo luciferase activity in wild-type and red-shifted luciferase modified trypanosomes. ONE-Glo luciferase activity expressed as signal to background in function of cell density (cells ml^−1^) (*T.b. brucei* AnTaR 1, *T.b. rhodesiense* RUMPHI, *T.b. gambiense* LiTaR 1 and *T.b. gambiense* 348 BT). Horizontal dotted line represents a fold change of 3. Vertical dotted lines mark the cell density necessary for detection at this threshold (5×10^3^ cells ml^−1^ for *T.b. brucei* AnTaR 1 P9 and approximately 10^5^ cells ml^−1^ for all CBR clones).
(TIF)

Figure S3 CellTiter-Glo luciferase activity in wild-type and red-shifted luciferase modified trypanosomes. CellTiter-Glo luciferase activity expressed as signal to background in function of cell density (cells ml^−1^) (*T.b. brucei* AnTaR, *T.b. rhodesiense* RUMPHI, *T.b. gambiense* LiTaR and *T.b. gambiense* 348BT). Horizontal dotted line represents a fold change of 3. The vertical dotted line marks the cell density necessary for detection at this threshold (approximately 10^4^ cells ml^−1^).
(TIF)

Table S1 List of primer and cDNA sequences and the resulting expression vector.
(DOCX)
