# Supplementary figures and images for: A Panel of Trypanosoma brucei Strains Tagged with Blue and Red-Shifted Luciferases for Bioluminescent Imaging in Murine Infection Models
Source: PLoS Negl Trop Dis. 2014 Aug 21;8(8):e3054. doi: 10.1371/journal.pntd.0003054 (PMC4140678; doi:10.1371/journal.pntd.0003054)

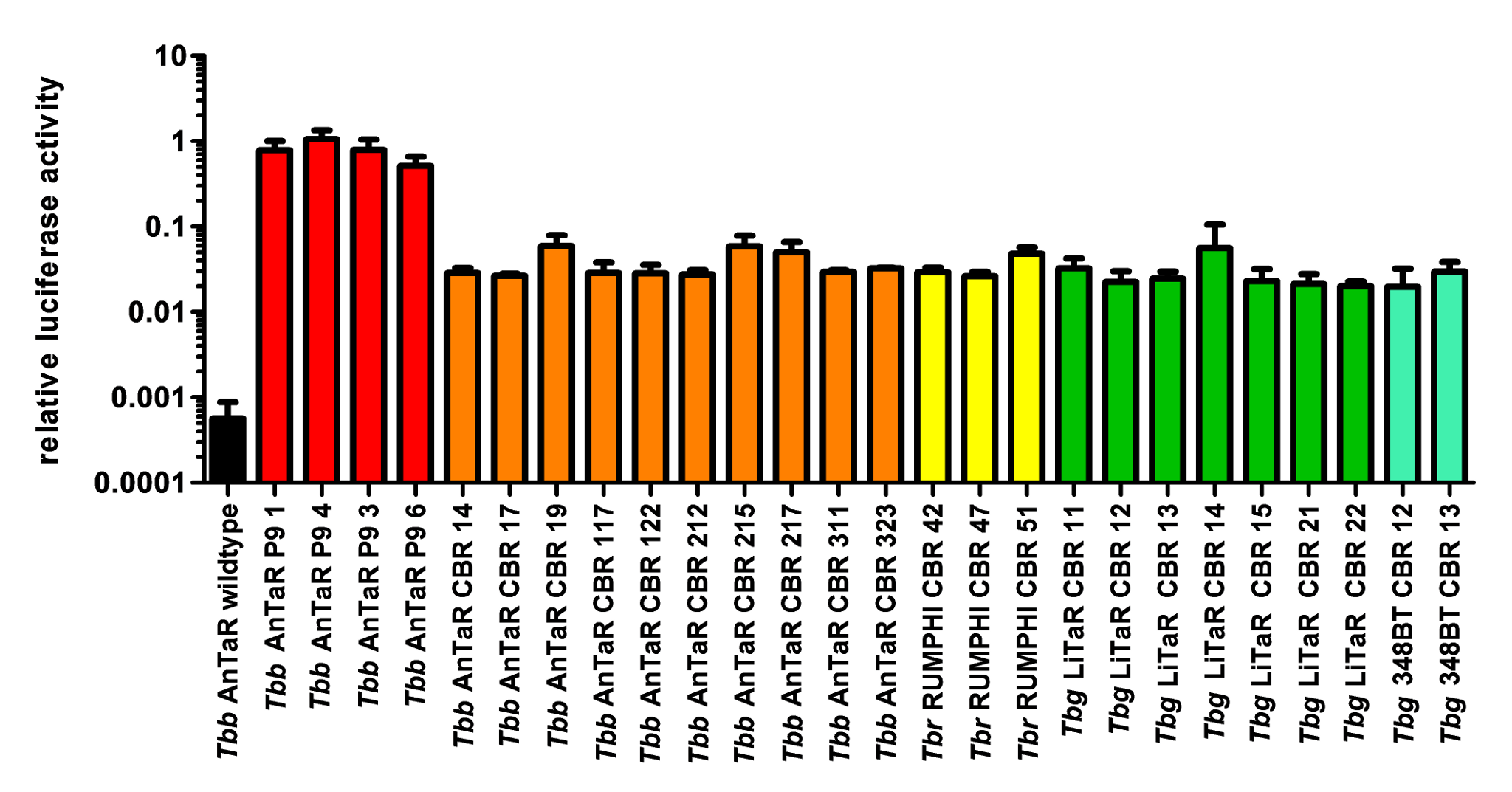

Supplement: Figure S1 — Relative luciferase activity of wild-type and red-shifted luciferase modified trypanosomes. Relative luciferase activity (mean of 2–8 repetitions ± SD) of the firefly luciferase modified clones of several strains (T.b. brucei AnTaR 1, T.b. rhodesiense RUMPHI, T.b. gambiense LiTaR 1 and T.b. gambiense 348 BT). (TIF) [file pntd.0003054.s001.tif]

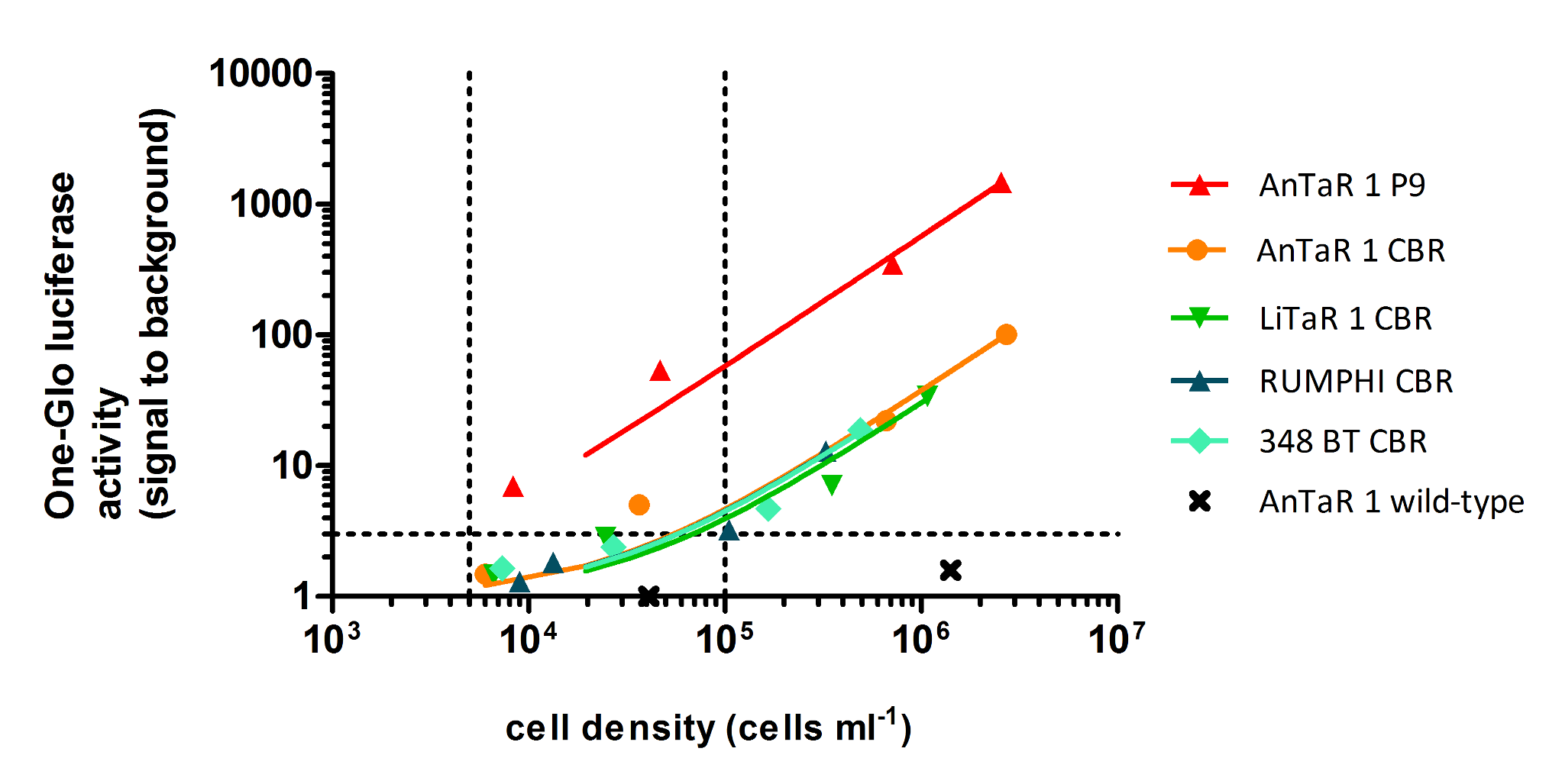

Supplement: Figure S2 — ONE-Glo luciferase activity in wild-type and red-shifted luciferase modified trypanosomes. ONE-Glo luciferase activity expressed as signal to background in function of cell density (cells ml−1) (T.b. brucei AnTaR 1, T.b. rhodesiense RUMPHI, T.b. gambiense LiTaR 1 and T.b. gambiense 348 BT). Horizontal dotted line represents a fold change of 3. Vertical dotted lines mark the cell density necessary for detection at this threshold (5×10^3^ cells ml−1 for T.b. brucei AnTaR 1 P9 and approximately 105 cells ml−1 for all CBR clones). (TIF) [file pntd.0003054.s002.tif]

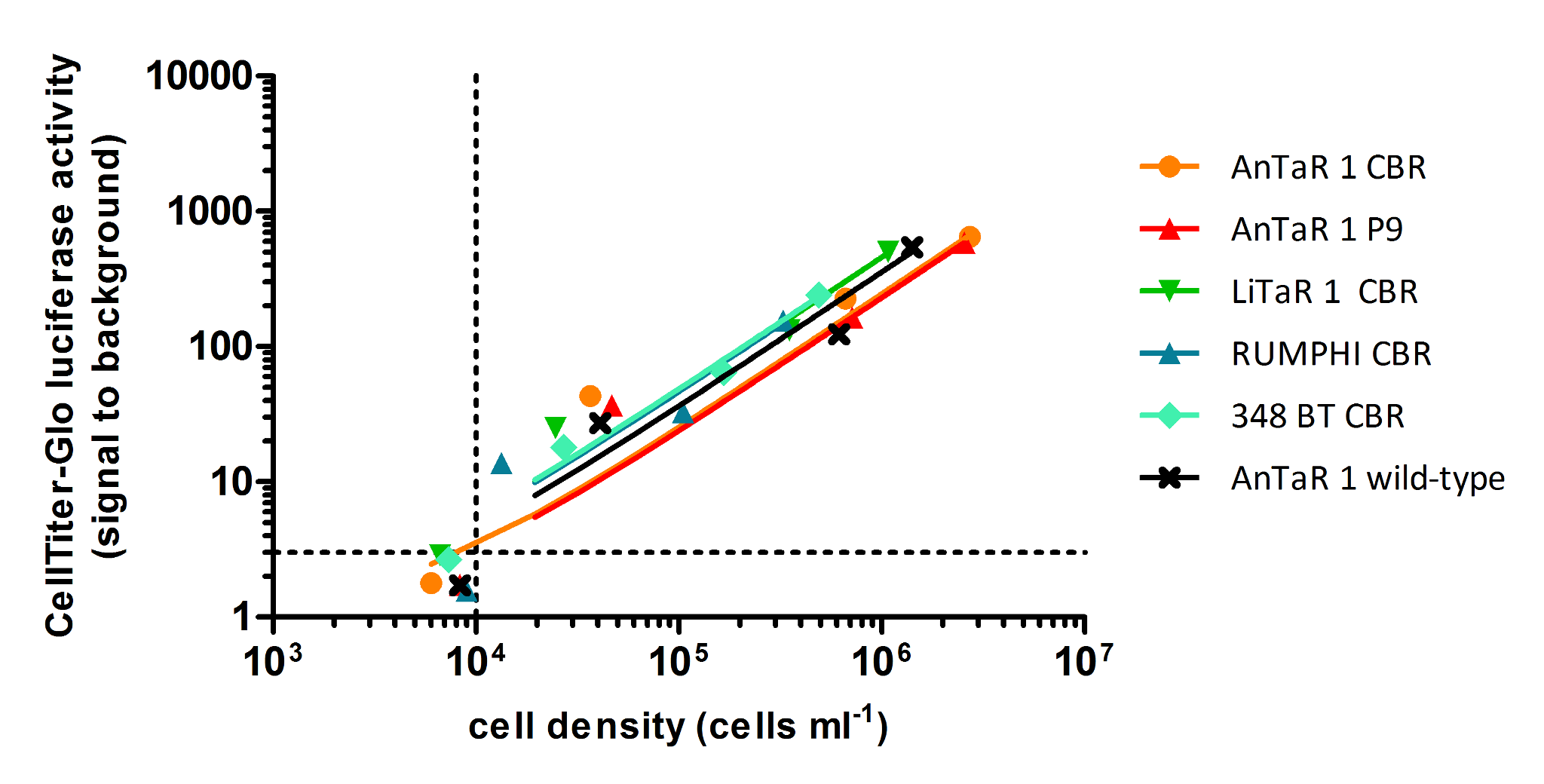

Supplement: Figure S3 — CellTiter-Glo luciferase activity in wild-type and red-shifted luciferase modified trypanosomes. CellTiter-Glo luciferase activity expressed as signal to background in function of cell density (cells ml−1) (T.b. brucei AnTaR, T.b. rhodesiense RUMPHI, T.b. gambiense LiTaR and T.b. gambiense 348BT). Horizontal dotted line represents a fold change of 3. The vertical dotted line marks the cell density necessary for detection at this threshold (approximately 104 cells ml−1). (TIF) [file pntd.0003054.s003.tif]
