# Supplementary material for: A Panel of Trypanosoma brucei Strains Tagged with Blue and Red-Shifted Luciferases for Bioluminescent Imaging in Murine Infection Models
Source: PLoS Negl Trop Dis. 2014 Aug 21;8(8):e3054. doi: 10.1371/journal.pntd.0003054 (PMC4140678; doi:10.1371/journal.pntd.0003054)
Supplement: Table S1 — List of primer and cDNA sequences and the resulting expression vector. (DOCX) [file pntd.0003054.s004.docx]

**Table S1:** List of primer and cDNA sequences and the resulting expression vector

| Trypanosomal vector | Primer | Sequence | cDNA | Origin | Reference |
| --- | --- | --- | --- | --- | --- |
| pHD CBR | CBR-F | *AACTGCAACG***AAGCTT**ATGGTAAAGCGTGAGAAAAAT | CBR | pCBR-Basic Vector | Promega |
|  | CBR-R | *TAAATGGGCA***GGATCC**CTAACCGCCGGCCTT | CBR | pCBR-Basic Vector | Promega |
| pHD P9 | P9-F | *AACTGCAACG***AAGCTT**ATGGAGGAC | PpyRE9 | pGEX-6P.2PpyRE9 | [37] |
|  | P9-R | *TAAATGGGCA***GGATCC**TCAGATCTTG | PpyRE9 | pGEX-6P.2PpyRE9 | [37] |

Italic: vector specific sequence. Bold: restriction site. Underlined: reporter specific sequence
